# Schmidt's Syndrome: An Uncommon Cause of Spontaneous Hypoglycemia

**DOI:** 10.1055/s-0044-1779745

**Published:** 2024-02-27

**Authors:** George Sarin Zacharia, Anu Jacob, Binu Mary Bose

**Affiliations:** 1 Department of Internal Medicine & Gastroenterology, Ahalia Hospital, Mussafah, Abu Dhabi, United Arab Emirates; 2 Department of Anaesthesiology, Ahalia Hospital, Mussafah, Abu Dhabi, United Arab Emirates; 3 Department of Pathology, Malankara Orthodox Syrian Church Medical College, Ernakulum, Kerala, India

**Keywords:** Schmidt's syndrome, APS-2, polyendocrine syndrome, Addison's disease, hypoglycemia, hypothyroidism

## Abstract

Schmidt's syndrome, or autoimmune polyendocrine syndrome type 2 (APS-2), is an uncommon disorder characterized by the co-occurrence of autoimmune thyroiditis and adrenalitis. APS-2 is defined as a combination of Addison's disease, autoimmune thyroid disease, and/or type 1 diabetes mellitus. It is an autosomal dominantly inherited polygenic disorder with incomplete penetrance; the candidate genes include but are not limited to HLA-DR3, HLA-DR4, CTLA-4, PTPN22, and CD25-IL-2. Autoimmune thyroiditis, often Hashimoto's disease, results in hypothyroidism. Primary adrenal failure results in enhanced secretion of adrenocorticotrophic hormone melanocyte and co-secretion of melanocyte-stimulating hormone, contributing to hyperpigmentation. Mineralocorticoid deficiency results in salt wasting, fatigue and cramps, postural hypotension, and hyperkalemia. Cortisol, an insulin counter-regulatory hormone, plays a pivotal role in maintaining euglycemia; deficiency predisposes to the development of hypoglycemia. We here report a rare presentation of Schmidt's syndrome as hypoinsulinemic hypoglycemia in a middle-aged male patient. Management includes treatment of acute hypoglycemic episodes with glucose or glucagon, long-term glucocorticoids and mineralocorticoids for adrenal insufficiency, and thyroid hormone supplements for hypothyroidism. This case report and brief overview aim to contribute to the scientific understanding of Schmidt's syndrome/APS-2. Additionally, here we briefly outline the diagnostic challenges in hypoglycemia evaluation, including the utilization of Whipple's triad and the gold standard supervised 72-hour fast and evaluation for primary adrenal and thyroid insufficiencies.

## Introduction

Named after Dr. Schmidt, who initially described the combination of lymphocyte-predominant thyroiditis and adrenalitis with consequential biglandular insufficiencies, in 1926, Schmidt's syndrome arises from the immune-mediated dysfunction of endocrine tissues. Almost four decades after this pioneering work, Carpenter et al 1 reported the frequent co-occurrence of this syndrome with type 1 diabetes mellitus, subsequently termed Carpenter's syndrome. Further studies confirmed the autoimmune pathophysiology, collectively reclassifying them as autoimmune polyendocrine syndrome type 2 (APS-2). This immune-endocrinopathy has a prevalence of 1:20,000 in the general population and is a polygenic inherited disorder with incomplete penetrance.

## Case Report

A 36-year-old male presented with complaints of excessive tiredness and fatigue for the last 1 year. He reported three episodes of loss of consciousness and sweating, informed to be due to hypoglycemia, and each episode improving with intravenous glucose therapy. He denied a history of diabetes mellitus or use of hypoglycemic agents or alcohol. The family history and review of systems were noncontributory. Physical examination revealed a thin middle-aged male with a body mass index of 16 kg/m ^2^ . The vital signs were normal except for bradycardia, which was 48 beats per minute. There was hyperpigmentation of buccal mucosa, palmar creases, knuckles, elbows, and knees. The thyroid was diffusely enlarged with no palpable nodules or cervical adenopathy. The hemogram was normal except for mild eosinophilia, 12%, with an absolute eosinophil count of 1,290 cells/mm ^3^ . Biochemistry revealed sodium of 132 mEq/dL and potassium of 5.6 mEq/dL, with normal renal and liver assays. Blood glucose levels were 64 mg/dL in fasting and 116 mg/dL postprandially. The fasting insulin and C-peptide levels were undetectable. A supervised 72-hour fast test was initiated; however, the patient developed symptomatic hypoglycemia at 13 hours of fasting. A blood sample was drawn, and the patient was administered intravenous glucose for correction of hypoglycemia. Upon analysis, the blood sugar was 41 mg/dL with undetectable insulin and C-peptide levels, consistent with hypoinsulinemic hypoglycemia.

**Fig. 1:**
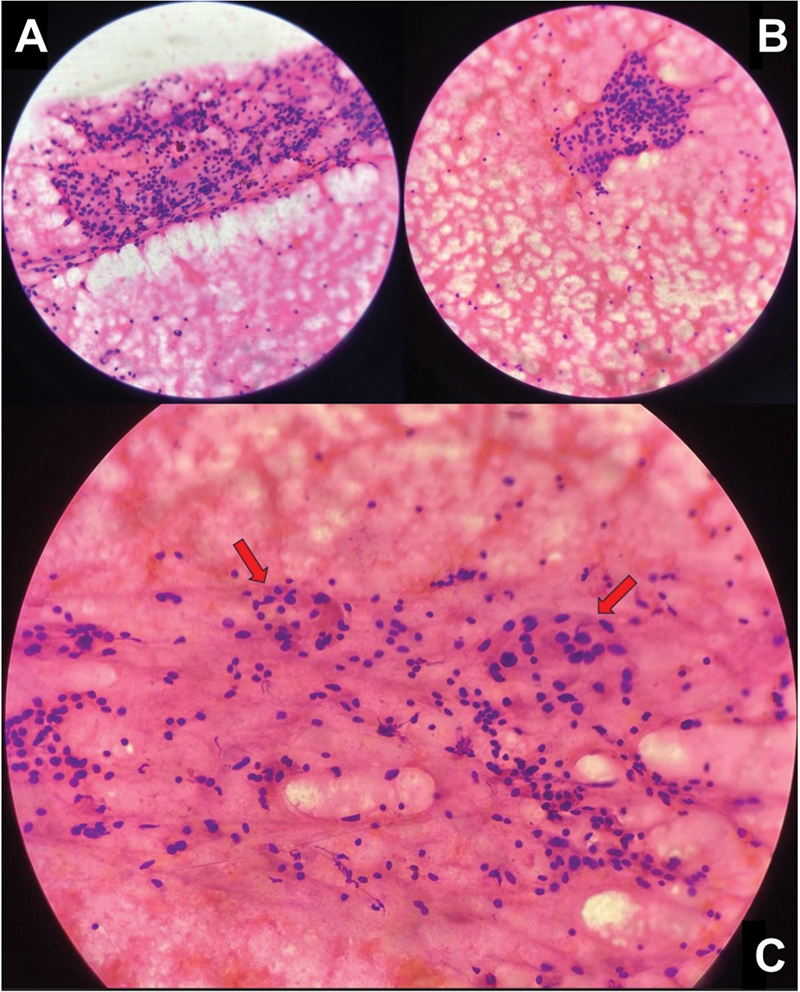
The hematoxylin and eosin-stained, microscopic images. ( **A** , **B** ) Thyroid follicular clusters with lymphocytic impingement. ( **C** ) Hürthle cell cluster with lymphocytic impingement (red arrows).

The serum thyroid panel was consistent with primary hypothyroidism: thyroid-stimulating enzyme (TSH) was 24 mIU/mL (0.35–4.50 mIU/mL), free triiodothyronine (fT3) was 1.1 pg/mL (2.4–4.2 pg/mL), and free thyroxine (fT4) was 0.3 ng/dL (0.8–1.7 ng/dL). The antithyroid peroxidase (TPO) antibodies were positive: 110 IU/mL (<0.3 IU/mL). The thyroid fine-needle aspiration cytology was typical for autoimmune thyroiditis ( Fig. 1 ).

The fasting cortisol level was <1 µg/dL (5–25 µg/dL). The cosyntropin test revealed a basal adrenocorticotropic hormone (ACTH) of 128 pg/mL (9–52 pg/mL), stimulated cortisol levels of 1.2 and 1.1 µg/dL at 30 and 60 minutes, respectively, consistent with primary adrenal insufficiency. The 21-hydroxylase antibodies were positive: 2.1 U/mL (<1 U/mL), suggesting autoimmune adrenalitis. Abdominal magnetic resonance imaging revealed small-to-normal-sized adrenal glands without evidence of necrosis, hemorrhage, mass, or calcification.

The diagnosis of Schmidt's syndrome was made due to the coexisting autoimmune thyroid and adrenal insufficiencies. Serum luteinizing hormone, follicle-stimulating hormone, prolactin, and testosterone measurements were normal. He was initiated on thyroid supplements and prednisolone. Upon follow-up, the patient showed marked improvement in his symptoms, no further hypoglycemic episodes, and normalization of serum electrolytes and thyroid profile. Antibodies to glutamic acid decarboxylase were tested during the follow-up visit and were reported undetectable.

## Discussion

Schmidt's syndrome, named after the German physician who initially described a biglandular disease with hypothyroidism and Addison's disease, is a rare autoimmune disorder. Subsequently, it was incorporated into APS-2, which is associated with a myriad of endocrinopathies, most frequently autoimmune thyroid disease, Addison's disease and type 1 diabetes mellitus and less frequently celiac disease, pernicious anemia, hypogonadism, myasthenia gravis, etc. 2 3 APS-2 is defined as a combination of Addison's disease, autoimmune thyroid disease, and/or type 1 diabetes mellitus. 4 The reported annual incidence of APS-2 is 1-2:100,000, more frequent in females than males, with a ratio of 3:1. 2 It is a polygenetic disorder with familial clustering of cases, inherited autosomal dominant with incomplete penetrance, linked to genes HLA-DR3, HLA-DR4, CTLA-4, PTPN22, CD25-IL-2, and more. 2 3 5 Autoimmune-mediated lymphocytic infiltration of the glands results in various endocrinopathies.

Whipple's triad refers to the criteria for the diagnosis of hypoglycemia: (i) symptoms of hypoglycemia, (ii) low blood glucose levels, and (iii) relief of symptoms upon correction of glucose levels. Symptoms include tremors, palpitations, sweating, confusion, seizures, loss of consciousness, coma, and even death. 6 7 The gold standard diagnostic test for spontaneous hypoglycemia is the supervised 72-hour fast test. 8 Low insulin and C peptide levels during hypoglycemia virtually rule out hyperinsulinemic hypoglycemia. Hypoinsulinemic hypoglycemia can result from excess alcohol consumption, malnutrition or starvation, and deficiency of insulin-antagonistic counter-regulatory hormones. The counter-regulatory hormones include glucagon, cortisol, adrenaline, and growth hormone. 9 Regardless of the etiology, hypoglycemic episodes should be promptly treated with glucose and or glucagon. If the blood sugar ranges between 55 and 70 mg/dL, the 15 to 15 rule is recommended; that is, administer 15 g of oral carbohydrates, recheck after 15 minutes, and repeat the steps until the blood glucose is more than 70 mg/dL. However, if the blood sugar is less than 55 mg/dL, the individual will require intravenous glucose, the initial dose of 25 grams, or glucagon, 1 mg, administered intravenously, intramuscularly, or subcutaneously. 8 10

Addison's disease, or primary adrenal insufficiency, most frequently results from autoimmune adrenalitis, while rarer causes include infections including tuberculosis, hemorrhage, tumors including metastasis, medications, and adrenal dystrophy. Clinical manifestations are often nonspecific and subtle, like anorexia, nausea, vomiting, fatigue, postural giddiness, hypotension, and hyperpigmentation involving but not limited to buccal mucosa, lips, nail beds, palmar creases, and nipples. Low serum cortisol and elevated ACTH, with minimal response to ACTH stimulation, are consistent with the diagnosis of primary adrenal dysfunction. Anemia, eosinophilia, and hyperkalemia with or without hyponatremia are diagnostic clues on routine blood workups in Addison's disease. Other endocrine abnormalities include low aldosterone, elevated plasma renin activity, and low dehydroepiandrosterone (DHEA) levels. A positive antibody to 21-hydroxylase suggests an autoimmune etiology, while adrenal imaging helps rule out most other adrenal diseases. Treatment includes a combination of glucocorticoids and mineralocorticoids. DHEA therapy might benefit individuals with sexual and mood disorders. 11

Autoimmune thyroid diseases can result in hypo or hyperfunctioning gland, hypothyroidism being more common. Hashimoto's thyroiditis is a form of autoimmune thyroiditis, typically resulting in primary hypothyroidism, and is the most common thyroid manifestation in Schmidt's syndrome or APS-2. Clinical manifestations include fatigue, constipation, weight gain, cold intolerance, bradycardia, nonpitting edema, and diffuse thyroid swelling. Primary hypothyroidism is characterized by an elevated TSH and low fT3 and fT4 levels. Anti-TPO antibodies are present in 95% of patients. 12 Thyroid sonography reveals a normal to bulky thyroid gland, which is hypo or heteroechoic with increased vascularity. Fine-needle aspiration cytology reveals lymphocyte predominant inflammatory infiltration, giant cells, Hürthle cell change, and follicular epithelial cells. Management strategy includes thyroid hormone supplements and monitoring.

Concomitant autoimmune adrenal and thyroid deficiencies in our patient are consistent with the diagnosis of Schmidt's syndrome or APS-2. The spontaneous hypoinsulinemic hypoglycemia most likely resulted from adrenal insufficiency. Schmidt's syndrome presenting as hypoglycemic is uncommon, though reported in the literature. 13 14 Most reported cases, however, have had concomitant type 1 diabetes mellitus, strikingly absent in our case. Our case highlights the need for thorough evaluation in cases of hypoinsulinemic hypoglycemia to rule out counter-regulatory hormone deficiencies.

## Conclusion

Schmidt's syndrome or APS-2 refers to a combination of Addison's disease, autoimmune thyroid disease, and/or type 1 diabetes mellitus. The constellation has many manifestations, corresponding to hormone deficiencies, but with nonspecific symptoms. Autoimmune thyroiditis typically culminates in hypothyroidism. Primary adrenal insufficiency manifests as hyperpigmentation, hyperkalemia, postural hypotension due to salt wasting, eosinophilia, and/or sexual dysfunction. Hypoglycemia is an uncommon manifestation of Schmidt's syndrome. Cortisol, an insulin counter-regulatory hormone, plays a pivotal role in maintaining euglycemia; deficiency predisposes to the development of hypoglycemia. This case report underscores the importance of diligent analysis and interpretation of endocrine data for diagnostic and therapeutic purposes in polyendocrine syndrome and hypoinsulinemic hypoglycemia.
